# Chemical Synthesis of Rare, Deoxy-Amino Sugars Containing Bacterial Glycoconjugates as Potential Vaccine Candidates

**DOI:** 10.3390/molecules23081997

**Published:** 2018-08-10

**Authors:** Archanamayee Behera, Suvarn S. Kulkarni

**Affiliations:** Department of Chemistry, Indian Institute of Technology Bombay, Mumbai 400076, India; archana@chem.iitb.ac.in

**Keywords:** bacterial glycoconjugates, rare deoxy-amino sugars, total synthesis, vaccine candidates, zwitterionic polysaccharides, stereoselective glycosylation

## Abstract

Bacteria often contain rare deoxy amino sugars which are absent in the host cells. This structural difference can be harnessed for the development of vaccines. Over the last fifteen years, remarkable progress has been made toward the development of novel and efficient protocols for obtaining the rare sugar building blocks and their stereoselective assembly to construct conjugation ready bacterial glycans. In this review, we discuss the total synthesis of a variety of rare sugar containing bacterial glycoconjugates which are potential vaccine candidates.

## Introduction

**Figure 1:**
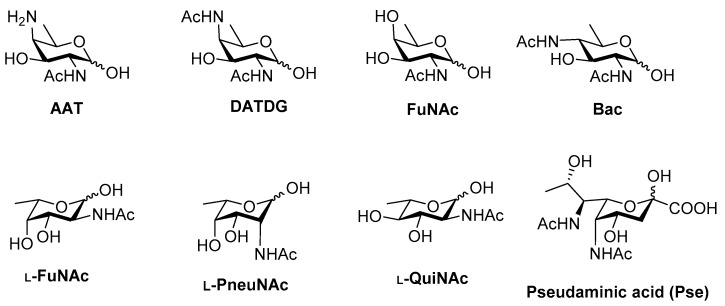
Representative examples of rare deoxy amino monosaccharides present in bacteria.

Carbohydrates in the form of glycoconjugates are ubiquitously distributed on the cell surface. By virtue of their position and unique structures, they play key roles in a myriad of vital life processes at the cell–cell interface [1]. For several years, it was believed that glycosylation, the post-translational modification of proteins, is present only in eukaryotes and that it is virtually absent in prokaryotes. However, it is now well established that bacteria and even archaea have glycans present on their surfaces as well. Several bacteria possess capsular polysaccharides (CPS) and other types of carbohydrates such as *O*-antigens, exopolysaccharrides and teichoic acids, which are immunogenic and hence useful for vaccine development [2]. Currently, several glycoconjugate vaccines are under investigation for various infectious diseases [3]. Very recently, Adamo and co-workers extensively reviewed the progress in the development of antimicrobial glycoconjugate vaccines with special emphasis on targets for future development [4]. An important aspect of bacterial glycoproteins and polysaccharides is that many of them possess unique rare deoxy amino monosaccharides which are virtually absent in humans (Figure 1). More importantly, their presence has been shown to be linked with pathogenesis [5,6,7]. Since these structures are not present in humans, such glycans are potential vaccine candidates and also useful tools for identification, detection and selective targeting of bacteria [8,9,10,11,12]. Over the past 15 years, efficient routes have been developed for the synthesis of rare monosaccharide building blocks starting from simple sugars or via *de-novo* approaches from amino acid derivatives. These approaches have been categorically reviewed and discussed by Kulkarni and co-workers [13]. In this article, we review the progress made in the chemical assembly of the rare deoxy amino sugars containing oligosaccharides which are potential candidates for future vaccine development.

## *Campylobacter jejuni* Heptasaccharide

*Campylobacter jejuni* is a Gram-negative pathogen. It contains a major non flagellin antigenic glycoprotein designated as PEB3 or Cj0289c [14]. This glycoprotein carries *N*-linked glycans and have multiple glycosylation sites [15]. The core structure of this glycan was established as α-Gal*p*NAc-(1→4)-α-Gal*p*NAc-(1→4)-[β-Glc*p*-(1→3)]-α-Gal*p*NAc(1→4)-α-Gal*p*NAc-(1→4)-α-GalpNAc-(1→3)-β-Bacp heptasaccharide. The oligosaccharide contains a rare sugar bacillosamine (2,4-diacetamido-2,4,6-trideoxy-d-glucopyranose), and repeating d-galactosamine building blocks with branched d-glucose. The presence of the *N*-linked glycan on the surface of *C. jejuni* has been shown to be crucial for pathogenesis, specifically in enteric adhesion to host cells, which is the first step of virulence [16,17]. *C. jejuni* causes gastroenteric disorders and is implicated in neuromuscular paralysis as well as Guillian Barre syndrome (GBS) [18]. The biosynthetic pathway of *C. jejuni* has been delineated by Imperiali and co-workers [19,20,21]. In 2006, Ito and co-workers reported the first chemical synthesis of the heptasaccharide [22]. The synthesis involves stereoselective formation of the repeating unit α-GalpNAc-(1→4)-α-GalpNAc motif, its assembly to construct the branched hexasaccharide Glc_1_GalNAc_5_ and coupling with bacillosamine.

**Scheme 1:**
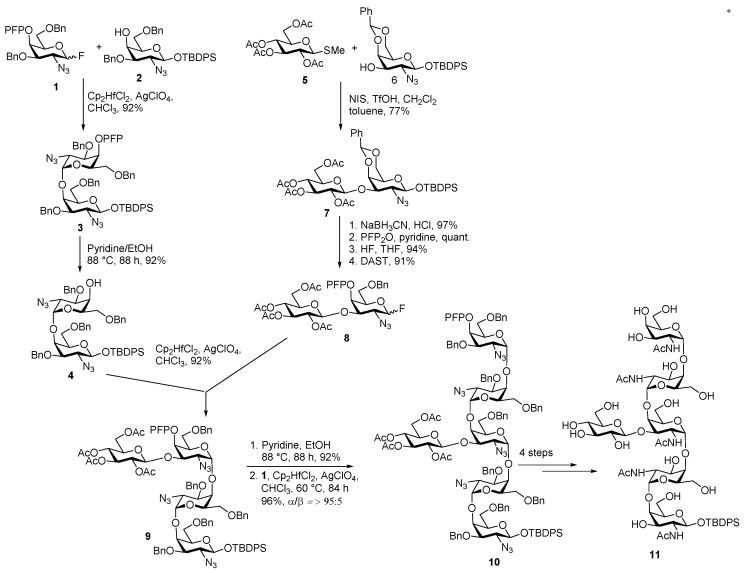
Ito’s first-generation synthesis of the heptasaccharide from *C. jejuni.*

The assembly of *C. jejuni* hexasaccharide is shown in Scheme 1. Coupling of glycosyl fluoride **1** with 4-OH galactosamine acceptor **2** in the presence of Cp_2_HfCl_2_, AgClO_4_ promoter delivered key disaccharide **3** in a highly stereoselective manner with excellent yield. The α-selectivity observed in this case is a combined effect of the anomeric effect, the remote participation of C4 pentafluoro propionyl (PFP) group and non-participating C2-azido group. Next, the PFP group was selectively removed under mild basic conditions using pyridine/EtOH at elevated temperature to afford disaccharide acceptor **4** in 92% yield. For the construction of the branched structure, glucopyranoside donor **5** was activated by NIS, TfOH activation condition and coupled with acceptor **6** to furnish the desired disaccharide donor (β-Glcp-(1→3)-GalpN_3_) **7** in 77% yield. Regioselective reductive O4 ring opening of benzylidine acetal, followed by capping of the free 4-OH with PFP group, anomeric desilylation and fluorination afforded glycosyl fluoride **8** in 91% yield. The so formed glycosyl fluoride **8** and disaccharide acceptor **4** were coupled together in the presence of Cp_2_HfCl_2_, AgClO_4_ condition at room temperature to obtain tetrasaccharide **9** in 92% yield. Similar deprotection of PFP group and further elongation furnished glucose branched hexasaccharide. Global deprotection was successfully conducted in a step wise manner to afford oligosaccharide **11** in good yield. 

**Scheme 2:**
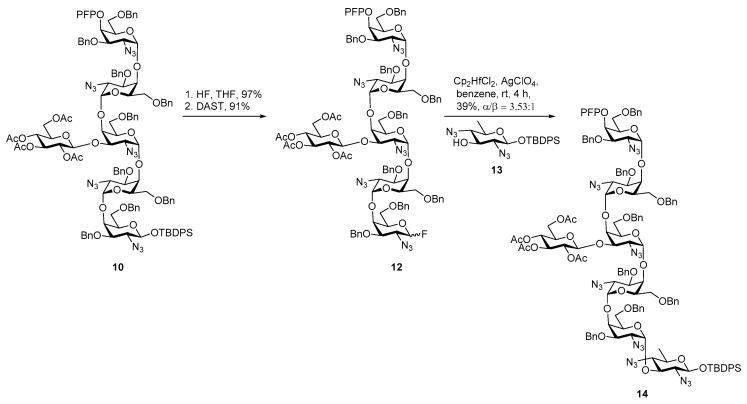
Glycosylation of *C. jejuni* hexasaccharide with bacillosamine.

As shown in Scheme 2, the final coupling of hexasaccharide **10** derived glycosyl fluoride **12** with bacillosamine building block **13** turned out to be inefficient, giving **14** in modest yield (39%) and low stereoselectivity (α/β = 3.53:1). Ito and co-workers also successfully coupled the bacillosamine building block with aspargine derivative to obtain Asn-linked Bac derivative [23]. 

**Scheme 3:**
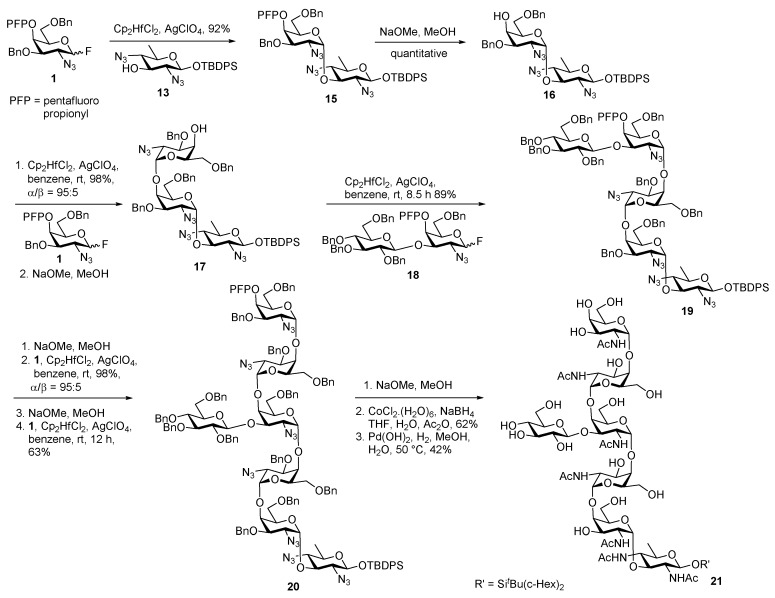
Ito’s second-generation total synthesis of *C. jejuni* heptasaccharide.

Subsequently, they improved the synthesis of heptasaccharide Glc_1_GalNAc_5_Bac_1_ by employing a linear glycosylation strategy for the assembly of glycan from reducing end to the non-reducing end (Scheme 3) [24]. The route involved stereoselective α glycosylation of di-azido-trideoxyglucose derivative **13** acceptor, with 4-*O*-PFP protected GalN_3_ donor **1** (GalN). AgClO_4_, Cp_2_HfCl_2_ promoted glycosylation of donor **1** with bacillosamine acceptor **13** gave α glycoside **15** in 92% yield. Deprotection of PFP group cleanly afforded 4′′-OH acceptor **16** which was glycosylated with galactosyl fluoride donor **1** under similar conditions at room temperature to give the trisaccharide adduct. Further PFP group cleavage using NaOMe, MeOH furnished trisaccharide acceptor **17** in 98% yield. Pentasaccharide **19** was assembled upon glycosylation of fluoro donor **18** with trisaccharide acceptor **17** in excellent yield with clean selectivity. Same deprotection and elongation sequence was carried out twice to get heptasaccharide **20**. Functional group deprotection was carried out carefully in a stepwise manner to get the target molecule **21**. 

## Zwitterionic Polysaccharides

Zwitterionic polysaccharides (ZPSs) form an important class of immunotherapeutic agents. Several ZPSs appear to stimulate distinct immunological responses that can activate a major histocompatability complex class II (MHCII)-mediated T-cell-dependent immune response in the absence of protein [25,26,27,28,29]. 

## ZPS of *Bacteroides fragilis*

PSA1 ZPS polysaccharide was isolated from the capsule of the commensal bacteria *Bacteroides fragilis* [30]. It shows anti-inflammatory properties and plays a key role in the development and the maintenance of a balanced mammalian immune system [31,32]. PSA1 stimulates IL-10 secretion, modulates surgical fibrosis [33], inhibits intestinal inflammatory disease caused by *Helicobacter hepaticus* [34] and protects against central nervous system (CNS) demyelinating disease [35].

**Scheme 4:**
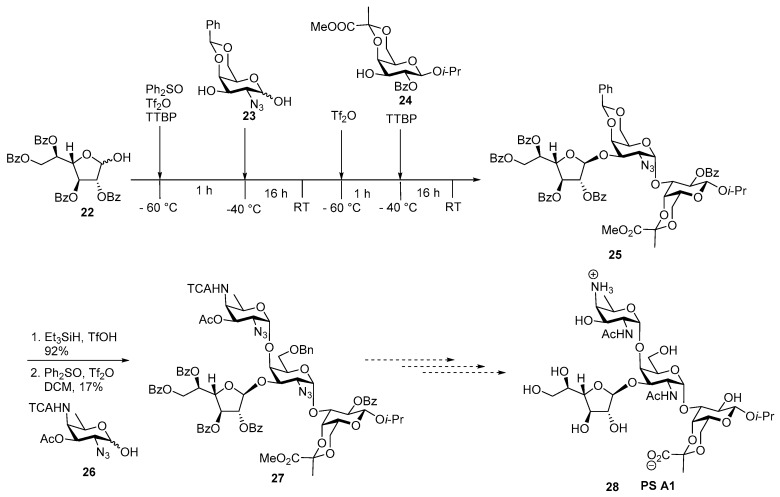
Van der Marel’s assembly of protected tetrasaccharide repeating unit of zwitterionic polysaccharides (ZPS) A1.

In 2007, van der Marel and co-workers reported a synthesis of the fully protected tetrasaccharide repeating unit of zwitterionic polysaccharide A1 (ZPS A1) as shown in Scheme 4 [36]. 

The major difficulties encountered in the assembly of tetrasaccharide are the synthesis of 2-acetamido-4-amino-2,4,6-trideoxy-d-galactopyranose AAT building block and its coupling to d-galactosamine unit through α (1→4) linkage. Trisaccharide **25** was synthesized in a one-pot manner using iterative dehydrative glycosylation conditions. Initially hemiacetal donor **22** was pre-activated by Ph_2_SO, Tf_2_O and coupled with acceptor **23** to afford the corresponding disaccharide donor which was again activated under prevalent conditions upon addition of Tf_2_O and glycosylated with acceptor **24** to furnish the required trisaccharide **25** in 62% yield in one-pot manner. A regioselective triethylsilane reductive ring opening of benzylidene acetal **25** afforded the desired 4-OH acceptor which upon subsequent coupling with the pre-activated AAT donor **26** afforded the tetrasaccharide repeating unit **27** of ZPS A1 in 17% yield. The low yield in the final step could be due to the mismatching reactivity of disarming AAT donor and sterically hindered trisaccharide acceptor. 

**Scheme 5:**
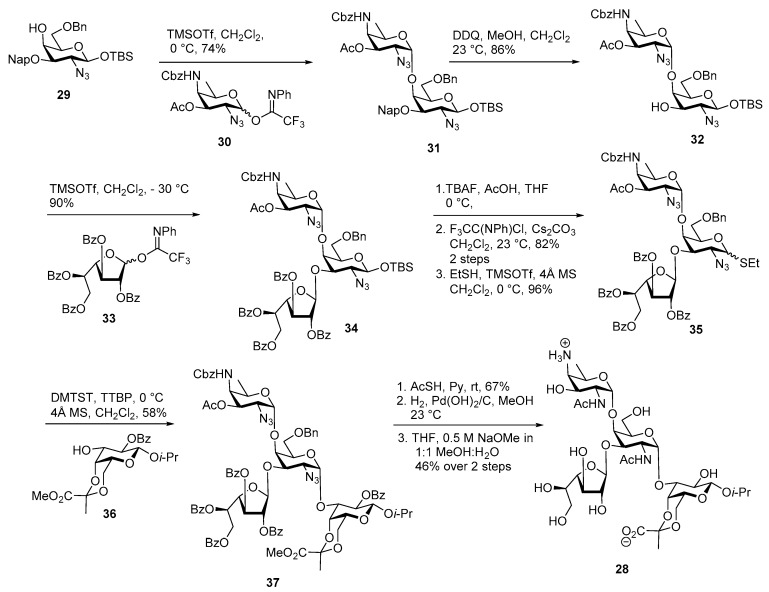
First total synthesis of ZPS A1 by Seeberger and co-workers.

In 2010, Seeberger and co-workers established a successful route to accomplish the total synthesis of ZPS A1 (Scheme 5) tetrasaccharide [37]. After trying several glycosylation routes, they concluded that the coupling of AAT donor with d-galactosamine acceptor has to be performed at the initial stage to get better coupling yields. Accordingly disaccharide **31** was assembled by coupling with AAT imidate donor **30** with d-galactosamine derived acceptor **29** using TMSOTf as a promoter in good yields with α/β ratio of 5:1 [38]. The AAT building block was synthesized via a *de novo* approach starting from commercially available l-threonine. Oxidative cleavage of 2-napthylmethyl group (Nap) using DDQ in CH_2_Cl_2_ and MeOH afforded the disaccharide acceptor **32** in 86% yield. Galactofuranose *N*-phenyl trifluoroacetimidate **33** was successfully coupled with the corresponding disaccharide acceptor **32** at −30 °C to assemble trisaccharide adduct **34** in 90% yield as a single isomer. The anomeric TBS group was cleaved using TBAF in AcOH to give its corresponding lactol and subsequently converted into *N*-phenyl trifluoroacetimidate in 82% yield over two steps.

The so-formed imidate donor was converted into its corresponding ethyl thioglycoside **35** for further glycosyation. Thioglycoside donor **35** was activated by DMTST, TTBP at 0 °C and coupled with galactose methyl pyruvate acceptor **36** to afford tetrasaccharide **37** in 58% yield as α isomer. Global deprotection was carried out over four steps to obtain tetrasaccharide repeating unit PSA1 of *Bacteroides fragilis*.

Later on in 2013, Kulkarni and co-workers synthesized the AAT containing key disaccharide of ZPS A1 in an efficient manner [39]. The rare sugar AAT **38** and d-galactosamine acceptor **39** were synthesized from commercially available d-mannose via nucleophilic displacements of 2,4-bis triflates in one pot manner [39]. 

**Scheme 6:**
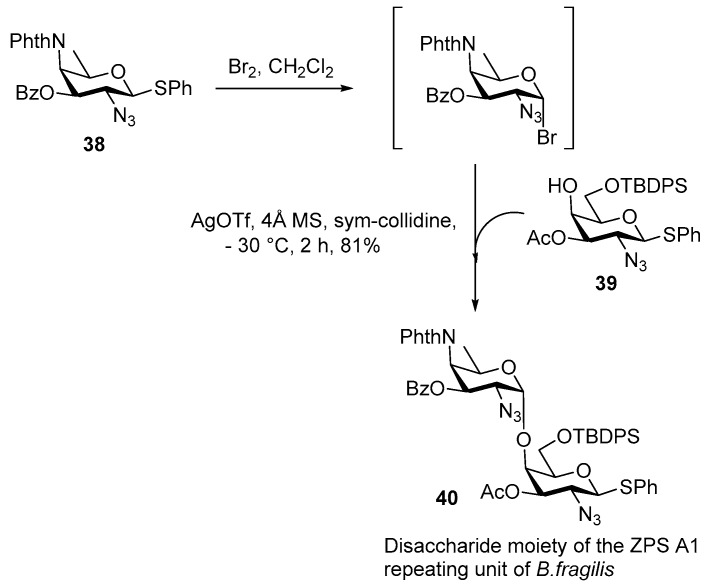
Kulkarni’s synthesis of the rare disaccharide donor unit of ZPS A1.

Thioglycoside AAT derivative **38** (Scheme 6) was converted to its corresponding glycosyl bromide in situ and treated with d-galactosamine acceptor **39** in the presence of AgOTf at −30 °C to give the disaccharide moiety **40** of ZPS A1 in 81% yield as a single α-isomer. The α-stereoselectivity in this case is perhaps arising through the possible formation of a more reactive β-glycosyl triflate using AgOTf and its subsequent S_N_2 type displacement by the nucleophilic 4-OH acceptor **39**. 

Andreana and co-workers employed a very novel and unique approach to use PSA1 tetrasaccharide for conjugation with other glycans such as the tumor associated cancer antigens Tn [40] and STn [41] as well as the repeating unit of *Streptococcus dysgalactiae* [42] 2023 polysaccharide, to construct PSA1 conjugate vaccines. Their studies show that attachment of PSA1 enhances the immune response of the respective glycans.

**Figure 2:**
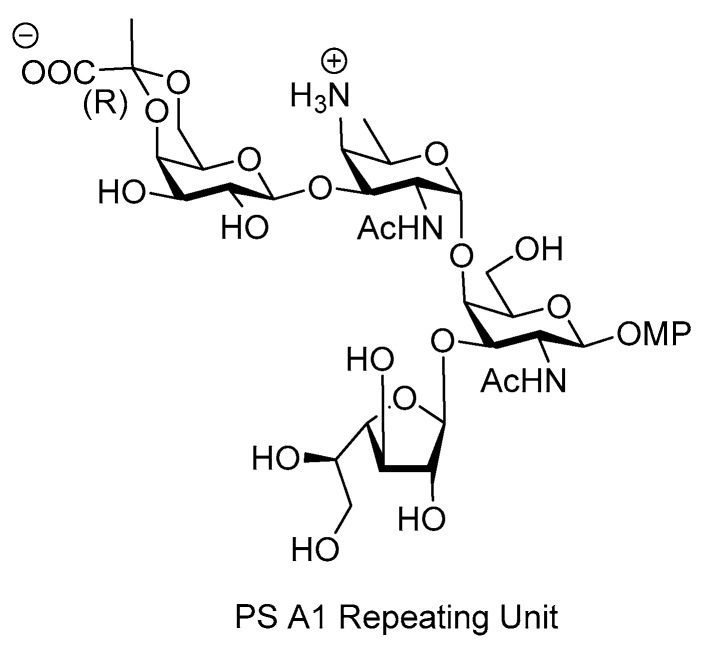
Structure of ZPS A1 with alternating charges on adjacent monosaccharides.

Very recently, Andreana and co-workers synthesized the PSA1 tetrasaccharide repeating unit with alternating charges on adjacent monosaccharides (Figure 2), employing a linear glycosylation strategy [43]. It is hoped that such a construct would display improved biological activity and its bio-evaluation is awaited.

## ZPS of *Streptococcus pneumoniae*

*Streptococcus pneumoniae* polysaccharides are another class of zwitterionic polysaccharides isolated by Wang et al. in 2002 [44]. *S. pneumoniae* is a Gram positive pathogen and consists of several layers of peptidoglycan to which teichoic acid and lipoteichoic acid are covalently attached. The bacteria causes a variety of life threatening diseases such as severe infection in upper respiratory tract, pneumonia, bacteremia and meningitis, thereby resulting in a high mortality rate [45,46,47]. It consists of a linear polymer of trisaccharide repeating units having a positively charged amino and two negatively charged carboxylic group, two galacturonic acids and a 2-acetamido-4-amino-2,4,6-trideoxygalactose residues. The major challenges in the synthesis of trisaccharides are low reactivity of uronic acid derivatives in glycosylation reactions, incorporation of successive α glycosidic bonds and late stage oxidation. Bundle and co-workers accomplished the first chemical synthesis of its ZPS trisaccharide repeating unit and the corresponding hexasaccharide containing two repeating units [48].

**Scheme 7:**
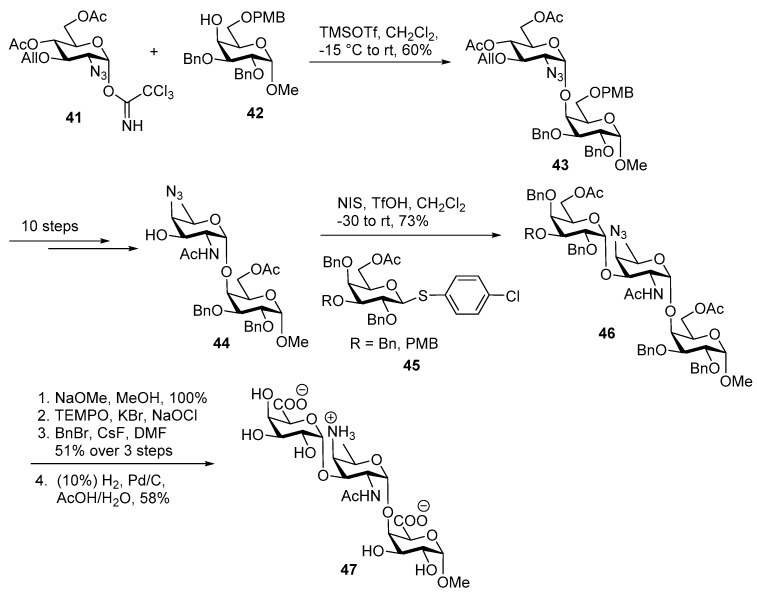
Bundle’s first total synthesis of *Streptococcus pneumoniae* ZPS trisaccharide.

As shown in Scheme 7, glycosylation of glucosamine imidate donor **41** with galactose 4-OH acceptor **42** under TMSOTf activation at −15 °C cleanly afforded α linked disaccharide **43** in 60% yield. After multiple steps, disaccharide **43** was deoxygenated at C6 and the *O*-allyl group C-3′ was cleaved via PdCl_2_-catalyzed reaction to afford the disaccharide acceptor **44** in moderate yield. Glycosylation of thioglycoside **45** under NIS/TfOH activation conditions in dichloromethane with acceptor **44** at −30 °C afforded the desired α-linked trisacchride **46** with good selectivity and a trace amount of β linked product was also detected. Global deprotection of **46** via selective deacetylation using NaOMe, MeOH, followed by oxidation of primary alcohol to benzyl esters using TEMPO, KBr, NaOCl and BnBr, CsF, DMF, followed by hydrogenolytic removal of benzyl groups furnished the target trisaccharide **47** in 58% yield. 

**Scheme 8:**
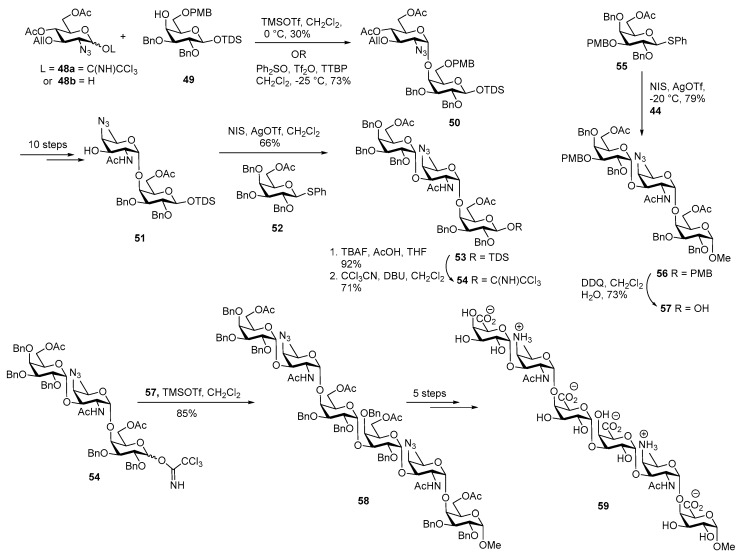
Bundle’s total synthesis of *Streptococcus pneumoniae* ZPS hexasaccharide.

Since the biological studies showed that trisaccharide did not activate T cell, Bundle and co-workers decided to synthesize hexasaccharide containing two repeating units as outlined in Scheme 8 [48]. The glucosamine imidate donor **48a** was coupled with galactose 4-OH acceptor **49** in the presence TMSOTf in CH_2_Cl_2_ at 0 °C to furnish α-linked disaccharide **50** in a low yield. In this case the 6-OAc group of donor **48a** is perhaps offering anchimeric assistance to obtain clean α-selectivity.

Alternatively, by using Gin’s dehydrative glycosylation strategy [49], diphenylsulfoxide and Tf_2_O mediated lactol activation of **48b** at −25 °C with acceptor **49** rapidly furnished only α-linked product **50** in 73% yield. Disaccharide acceptor **51** was synthesized following the same ten steps sequence that was employed for construction of disaccharide **44**. Coupling of galactose donor **52** and acceptor **51** using NIS, AgOTf promoter afforded desired trisaccharide **53** in 66% yield. The anomeric TDS group was removed by using TBAF in AcOH, followed by conversion to its corresponding imidate **54** in 71% yield. In parallel, galactose thiodonor **55** was glycosylated with disaccharide acceptor **44** at −20 °C to cleanly afford trisaccharide **56** in good yield. Then PMB group was selectively cleaved by using DDQ, CH_2_Cl_2_, H_2_O to provide trisaccharide acceptor **57** in 73% yield. Having both trisaccharide donor **54** and acceptor **57**, imidate donor **54** was activated by TMSOTf promoter to couple with acceptor **57** to obtain hexasaccharide **58** in 85% yield. Global deprotection afforded hexasaccharide repeating unit **59** of *Streptococcus pneumoniae*. 

**Scheme 9:**
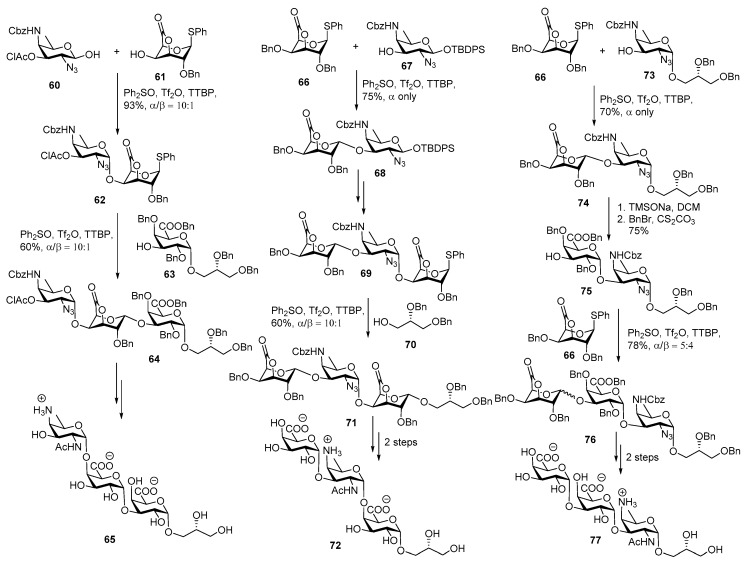
Codée’s synthesis of all the *Streptococcus pneumoniae* Sp1 ZPS trisaccharides.

In 2010, Codée and co-workers successfully synthesized all possible trisaccharide repeating units of the type 1 capsular polysaccharide of *Streptococcus pneumoniae* Sp1 [50]. For the synthesis of the first trisaccharide repeating unit (Scheme 9), the coupling of lactone **61** and 2,4,6-trideoxy-4-amino-d-galactosamine **60** using Ph_2_SO, Tf_2_O as a promoter afforded disaccharide **62** in α/β (10:1) ratio. Glycosylation between donor **62** and acceptor **63** using dehydrative glycosylation condition furnished fully protected glycerol capped trisaccharide **64** in 60% yield. The chloroacetyl group was removed, azide was reduced to NHAc, and subsequent debenzylation under hydrogenation conditions delivered the target trisaccharide **65** in 38% yield over two steps. For construction of trisaccharide **72**, lactone donor **66** was glycosylated with AAT 3-OH acceptor **67** using in situ generated diphenylsulfonium bistriflate, to afford disaccharide **68** in good yield and excellent stereoselectivity. Then lactone **68** was modified to its corresponding imidate donor through a multiple steps sequence and coupled with lactone acceptor to provide trisaccharide **69**. Pre-activation of trisaccharide thio donor **69** using Ph_2_SO, Tf_2_O and coupling with glycerol acceptor **70** furnished fully protected trisaccharide **71** in 60% yield (α/β = 10:1). 

The same deprotection strategy was applied to get the trisaccharide **72** in good yield as delineated in scheme 9.

For the synthesis of trisaccharide **77** (Scheme 9), disaccharide **74** was produced in a completely α-selective fashion in 70% yield. Lactone ring was opened using TMSONa and subsequent benzyl ester formation using BnBr and Cs_2_CO_3_, afforded disaccharide acceptor **75** in 75% yield over 2 steps. Then lactone donor **66** was coupled with disaccharide acceptor **75** using dehydrative glycosylation conditions to afford trisaccharide **76** in (α/β = 5:4) ratio. Global deprotection was carried out by reduction of azide to NHAc, lactone hydrolysis using TMSONa and hydrogenolysis using H_2_/Pd to remove benzyl groups to obtain target trisaccharide **77** in good yield.

**Scheme 10:**
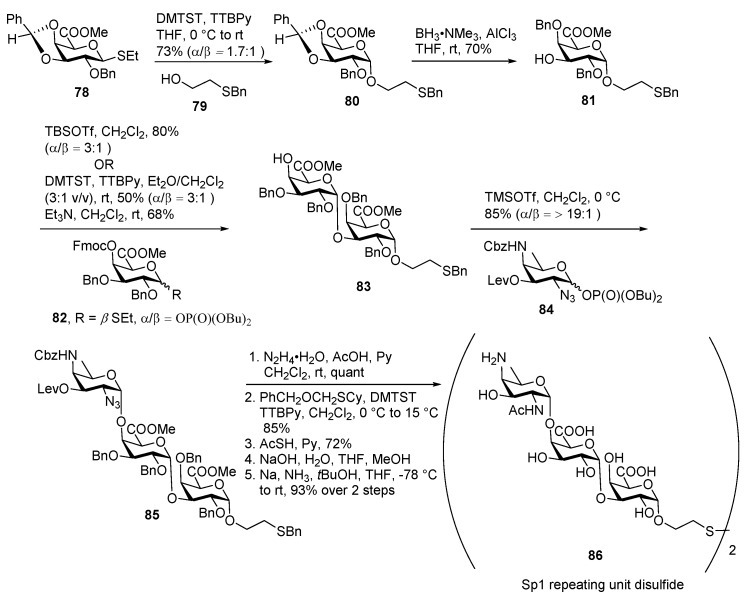
Seeberger’s total synthesis of thioether linked *S. pneumoniae* Sp1 trisacharide.

In 2014, Seeberger and co-workers reported a total synthesis of thioether linked trisaccharide repeating unit of *Streptococcus pneumoniae* Sp1 (Scheme 10) and its immunological characterization for the first time [51]. They positioned the AAT sugar at the non-reducing end to get a more effective immune response.

Thioether donor **78** was activated by DMTST in THF at 0 °C and coupled with thioether containing alcohol **79** to give glycoside **80** in 73% yield. Regioselective reductive ring opening of the endo-benzylidine ring using BH_3_·NMe_3_ and AlCl_3_ provided 3-OH acceptor **81** in good yield. Coupling of acceptor **81** with glycosyl phosphate donor **82**, resulted in disaccharide **83** as a mixture of diastereomers (α/β = 3:1) in 80% yield. To improve the ratio of diastereomers DMTST mediated glycosylation was performed on thio donor by using Et_2_O as a participating solvent. However, this also presented similar selectivity. Subsequent Fmoc group cleavage delivered disaccharide acceptor **83** in good yield. Trisaccharide **85** was obtained upon glycosylation of AAT phosphate donor **84** with disaccharide acceptor **83** using TMSOTf as a promoter in 85% yield as a single isomer. Fully protected trisaccharide **85** underwent deprotection steps to afford target molecule SP1 repeating unit disulfide **86**.

The Seeberger group also showed that a monovalent ST1 trisaccharide with the rare AAT sugar positioned at the non-reducing end induced a strong antibacterial immune response in rabbits and outperformed the ST1 component of the multivalent blockbuster vaccine Prevenar 13, leading to a more efficacious vaccine [52]. 

## Lipoteichoic Acid of *Streptococcus pneumoniae*

**Figure 3:**
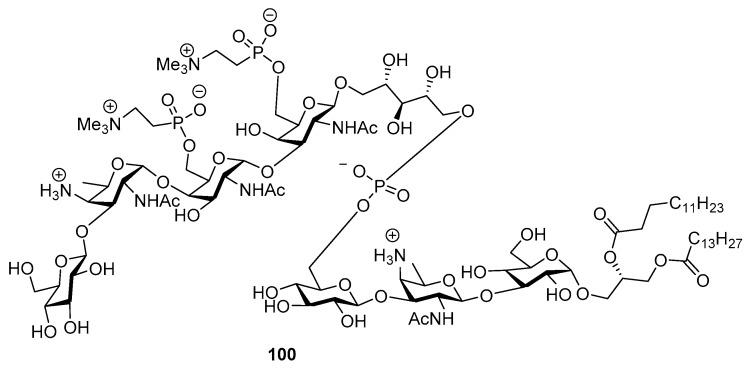
Structure of lipoteichoic acid of *S.pneumoniae.*

*Streptococcus pneumoniae* lipoteichoic acid (LTA) is a complex glycophospholipid that consists of nine glycan residues: three glucose, two galactosamine and two 2-acetamino-4-amino-2,4,6-trideoxygalactose (AATDgal) residues that are each differently linked, one ribitol and one diacylated glycerol (DAG) residue (*vide infra* Figure 3). It’s structural elucidation revealed that pneumococcal LTA of the R6 strain contains phosphodiester inter linked pseudopentasaccharide repeating units carrying each two phosphocholine residues and a glycolipidic core structure comprising a trisaccharide linked to diacylglycerol [53,54,55]. Its unprecedented structure and biological importance makes it more attractive to synthesize. Schmidt and co-workers reported its first chemical synthesis in 2010 [56].

**Scheme 11:**
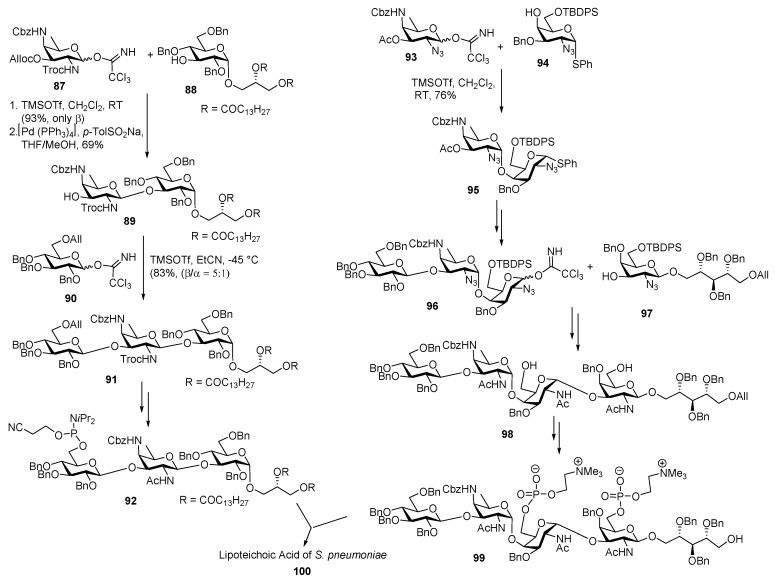
Schmidt’s total synthesis of lipoteichoic acid of *Streptococcus pneumoniae.*

For the synthesis of trisaccharide linked to diacylglycerol, AAT imidate donor **87** was coupled with glycerol derivative acceptor **88** in the presence of TMSOTf promoter to deliver β linked product in high yield. The Alloc group was selectively cleaved using palladium complex to afford disaccharide acceptor **89** in 69% yields. Reaction of **89** with glucose imidate donor **90** was performed under TMSOTf catalysis at −45 °C in propionitrile as a participating solvent to afford the desired β linked trisaccharide intermediate **91** with selectivity of 5:1 β*/*α ratio. This trisaccharide fragment was also synthesized by the same group, specifically to study its biological property [57]. Phosphorylation of this trisaccharide with bis(diisopropylamino)cyanoethoxyphosphine in the presence of diisopropylammonium tetrazolide furnished phosphorylated trisaccharide **92** in good yield as outlined in Scheme 11.

Pseudopentasaccharide, was constructed from pseudodisaccharide **97** as glycosyl acceptor and trisaccharide **96** as the glycosyl donor. AAT imidate donor **93** was glycosylated with 4-OH galactose acceptor **94** in the presence of TMSOTf promoter in CH_2_Cl_2_ at room temperature to obtain α-linked disaccharide **95** (due to NHCbz participation) which was further coupled with glucose donor to give trisaccharide derivative. Then the trisaccharide intermediate was converted to its corresponding imidate donor **96** and glycosylated with disaccharide acceptor **97** to afford pseudopentasaccharide **98** after multiple steps. Then phosphorylation was introduced using cholinoxy-cyanoethoxy-diisopropylaminophosphine using tetrazole as the activator, and then oxidation using *tert*-butyl hydroperoxide to furnish desired product **99** in high yield. The trisaccharide intermediates **92** and pseudopentasaccharide **99** were treated with tetrazole to deliver phosphite triester which further oxidized with tert-butyl hydroperoxide to give phosphate. The corresponding phosphate upon treatment with dimethylamine led to removal of the cyanoethyl group and formation of the desired phosphodiester dimethylammonium salt, which upon further global deprotection afforded target molecule of lipoteichoic acid of *S. pneumoniae* **100** (Figure 3). 

## CPS of *Streptococcus pneumoniae* Serotype 4

*S. pneumoniae* serotype 4 (ST4) CPS was discovered in 1931; the structure of its repeating unit was assigned only in 1988 [58,59,60,61,62,63]. This CPS is included in the commercial blockbuster vaccine Prevnar 13. The ST4 polysaccharide consists of a tetrasaccharide repeating unit made up of [3)-β-d-ManpNAc-(1→3)-α-l-FucpNAc-(1→3)-α-d-GalpNAc-(1→4)-α-d-Galp-2,3-(S)-Pyr-(1→]. The presence of *N*-acetyl sugars, an acid labile trans-2,3-(S)-pyruvate, β-mannoside and α-glycosidic linkages make this molecule a challenging synthetic target. The trisaccharide β-d-Man*p*NAc-(1→3)-α-l-Fuc*p*NAc-(1→3)-α-d-Gal*p*NAc, which lacks the pyruvalated galactose, has already been synthesized [64]. 

**Scheme 12:**
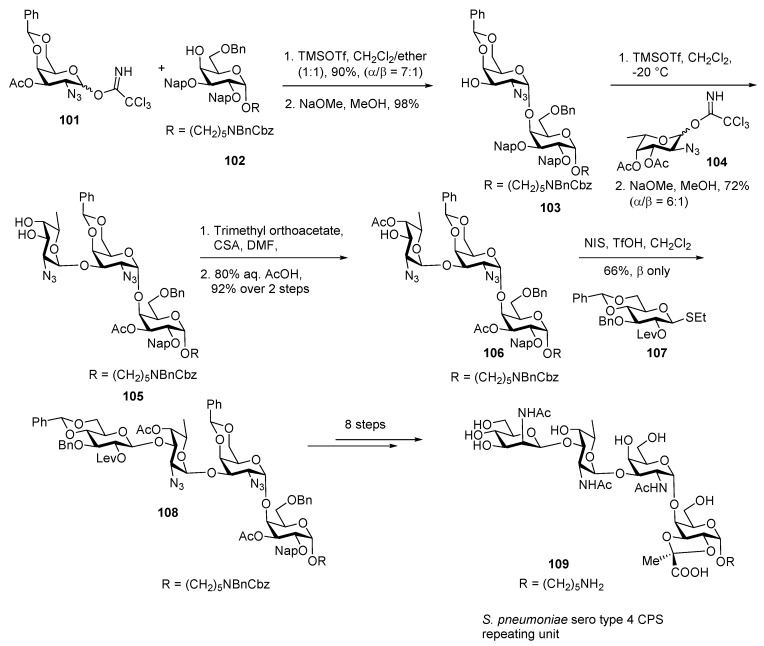
Seeberger’s total synthesis of capsular polysaccharides (CPS) repeating unit of *S. pneumoniae* Serotype 4.

In 2015, Seeberger and co-workers reported its first chemical synthesis as described in Scheme 12 [65]. Disaccharide can be assembled from galactosamine donor **101** with 4-OH acceptor **102** using TMSOTf promoter and ether as participating solvent in 90% yield with α/β ratio 7:1. Acetate group was cleaved using NaOMe, MeOH to afford acceptor **103** in good yield.

Glycosylation of acceptor **103** with l-fucosamine imidate donor **104** in the presence of TMSOTf promoter afforded a trisaccharide, which upon deacetylation delivered diol **105** in 72% yield a diastereomeric mixture (α/β = 6:1). Orthoester formation of diol **105**, and selective orthoester opening generated trisaccharide acceptor **106** in excellent yield. The tetrasaccharide **108** was obtained by coupling of trisaccharide acceptor **106** with glucose donor **107** using NIS, TfOH promoter which furnished only β adduct **108** in 66% yield. The gluco configuration of terminal sugar was converted to its corresponding manno configuration and subsequent global deprotection carried out in sequential manner delivered *S. pneumoniae* serotype 4 repeating unit tetrasaccharide **109**.

## *Streptococcus pneumoniae* Serotype 12F CPS

**Scheme 13:**
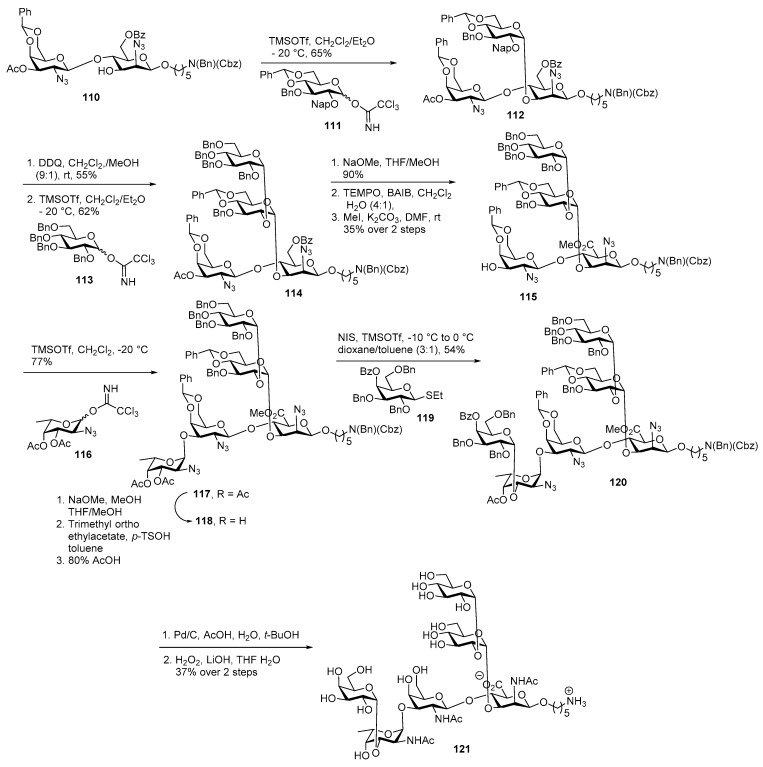
Seeberger’s total synthesis of *S. pneumoniae* serotype 12F CPS repeating unit.

Out of more than ninety, around twenty-three of the *S. pneumoniae* serotypes are responsible for about 90% of infections worldwide [66]. The licensed polysaccharide vaccine Pneumovax 23 contains serotype 12F. It is not efficacious in young children or elderly people, those at highest risk. The *S. pneumoniae* serotypes dominates with 85% of pneumococcal disease [67]. This CPS consists of hexasaccharide repeating units containing the [→4)-α-l-Fuc*p*NAc-(1→3)-β-d-Gal*p*NAc-(1→4)-β-d-Man*p*NAcA-(1→] polysaccharide backbone with a disaccharide branch at C3 of β-d-Man*p*NAcA and C3 of α-l-Fuc*p*NAc [68]. In 2017, Seeberger and co-workers reported chemical synthesis of *Streptococcus pneumoniae* serotype 12F CPS repeating unit employing a stepwise glycosylation route (Scheme 13) [69]. α-Selective glycosylation of disaccharide acceptor **110** with glucose imidate donor **111** using TMSOTf as the activator and diethyl ether as a participating solvent proceeded to produce trisaccharide **112** in 65% yield. Oxidative cleavage of C2 napthylmethyl group using DDQ, in CH_2_Cl_2_, MeOH delivered trisaccharide acceptor, which was subsequently treated with glucose imidate donor **113** in the presence of TMSOTf promoter and ether as a co-solvent at −20 °C to furnish tetrasaccharide **114** in 62% yield. 2-Azidomannose moiety of **114** was converted to the corresponding mannosaminuronic acid by cleaving the C6 benzoate ester using sodium methoxide in methanol and selective oxidation of the primary alcohol using BAIB/TEMPO followed by methyl ester formation to furnish tetrasaccharide acceptor **115** in 35% over 2 steps. TMSOTf promoted glycosylation of fucosamine donor **116** with acceptor **115** delivered pentasaccharide **117** as α-isomer by virtue of remote participation of the 4-*O*-acetate group. Acetate groups were cleaved by using NaOMe, MeOH, followed by orthoester formation and ring opening with 80% AcOH to afford pentasaccharide acceptor **118** in good yield. The desired hexasaccharide **120** was obtained as the α-anomer in 54% yield by coupling galactose building block **119** to pentasaccharide **118** using NIS/TMSOTf in a mixture of toluene/dioxane. Again, the C4 benzoate ester of **119** ensured high selectivity for the desired *cis*-glycosidic linkage. Global deprotection was carried out carefully to deliver target molecule **121**.

## CPS of *Streptococcus pneumoniae* Serotype 5

**Scheme 14:**
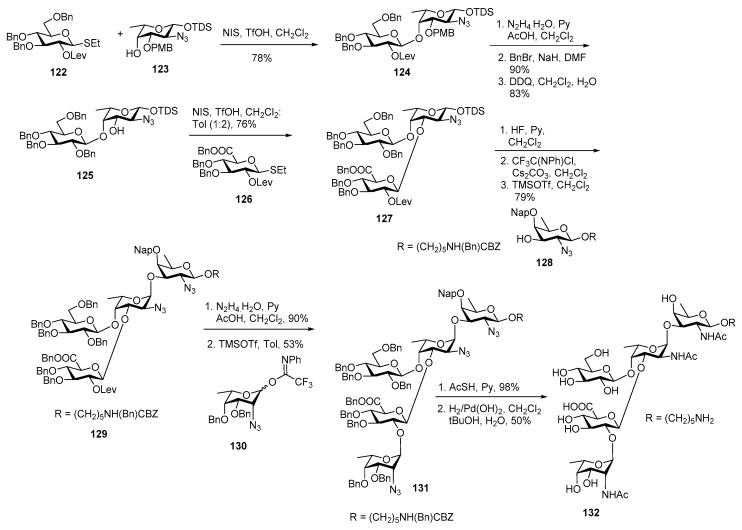
Seeberger’s synthesis of *S. pneumoniae* serotype 5 glycan.

*S. pneumoniae* serotype 5 (ST-5) is the fifth most prevalent *S. pneumoniae* serotypes with different CPS which causes invasive pneumococcal disease among young children globally [70,71]. The ST-5 repeating unit structure was assigned in 1985 [72]. It contains a central *N*-acetyl l-fucosamine (l-FucNAc) amino sugar that is linked to d-glucose at C4 and to d-glucuronic acid at C3, two rare deoxyamino sugars, the ketoamino sugar 2-acetamido-2,6-dideoxy-d-xylose-hexos-4-ulose (Sug*p*) and *N*-acetyl-l-pneumosamine (l-Pneu*N*Ac) as shown in Scheme 14. In 2017 Seeberger and co-workers reported chemical synthesis of the reduced form of the pentasaccharide repeating unit of *S. pneumoniae* serotype 5 [70]. 

Disaccharide **124** was furnished upon coupling of glucose donor **122** with l-fucosamine 4-OH acceptor **123** in the presence of NIS, TfOH promoter in 78% yield (Scheme 14). 

Levulinoyl group was deprotected using N_2_H_4_·H_2_O, subsequently 2′-OH was benzylated and PMB group at O2 position was selectively cleaved to afford disaccharide acceptor **125** in 83% yield. Trisaccharide **127** was assembled from glucuronic donor **126**, by coupling with acceptor **125** in the presence of NIS, TfOH promoter in 76% yield. The anomeric TDS group was cleaved using HF·Py to reveal the hemiacetal which was subsequently converted to its corresponding imidate donor which upon further glycosylation with d-fucosamine acceptor **128** furnished tetrasaccharide **129** in 79% yield. Selectively levulinoyl group was removed to obtain tetrasaccharide acceptor which was further coupled with l-pneumosazide donor **130** in toluene as a solvent to obtain fully protected α-linked pentasaccharide **131** in good yield. Global deprotection was carried out to deliver target molecule **132**.

## ZPS of *Shigella sonnei*

*Shigella* are Gram-negative enteroinvasive bacteria generally found in developing countries and industrial areas [73]. It mainly causes shigellosis in humans [74] which continues to be one of the five major diarrheal diseases in children under five [75]. 

The ZPS of *S. sonnei* has a disaccharide repeating unit made up of two uncommon aminosugars, a 2-acetamido-2-deoxy-l-altruronic acid (residue A) and a 2-acetamido-4-amino-2,4,6-trideoxy-d-galactopyranose (AAT, residue B) which are 1,2-trans linked to one another [76]. Mulard and co-workers accomplished chemical synthesis of all the ZPS oligosaccharides of *Shigella sonnei* [76]. 

## Synthesis of Disaccharide AB-Pr (1)

**Scheme 15:**
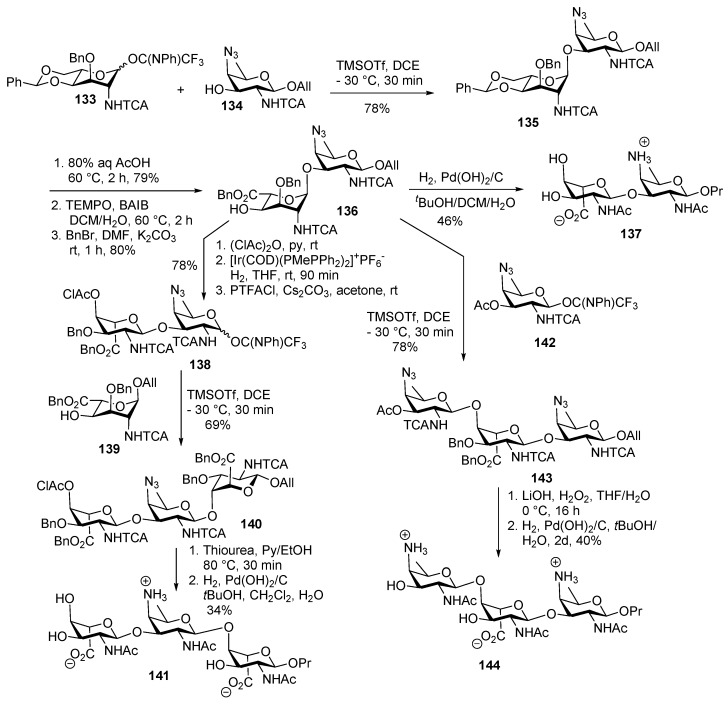
Mulard’s total synthesis of ZPS of *Shigella sonnei.*

AAT acceptor **134** and l-altrosaminyl donor **133** were coupled in the presence of TMSOTf at −30 °C to furnish disaccharide **135** (Scheme 15). Benzylidene cleavage followed by regioselective 6-OH oxidation by TEMPO, BAIB and benzyl ester protection of free acid using BnBr, NaHCO_3_ provided the disaccharide acceptor **136** in 80% yield over 2 steps. All benzyl groups were removed using H_2_/Pd(OH)_2_/C to deliver target disaccharide **137** in moderate yield.

Free 4′-OH of **136** was protected with chloroacetyl group, and subsequent cleavage of anomeric *O*-allyl group furnished the corresponding hemiacetal which was treated with PTFACl, Cs_2_CO_3_ to deliver disaccharide imidate donor **138** in 78% yield over 2 steps. For the synthesis of trisaccharides ABA′-Pr (2) and B′AB-Pr (3) disaccharide acceptor **136** and donor **138** were used. Uronate acceptor **139** was glycosylated with disaccharide imidate donor **138** at −30 °C to obtain fully protected trisaccharide **140** as a sole product. Chloroacetyl group was cleaved using thiourea, pyridine followed by hydrogenolysis to afford trisaccharide **141**. TMSOTf-mediated coupling of acceptor **136** with the AAT donor **142** resulted in trisaccharide **143** in 78% yield. Global deprotection was carried out to deliver trisaccharide **144** in good yield over 2 steps.

## Phosphorylated ZPS of *Providencia alcalifaciens* O22

**Scheme 16:**
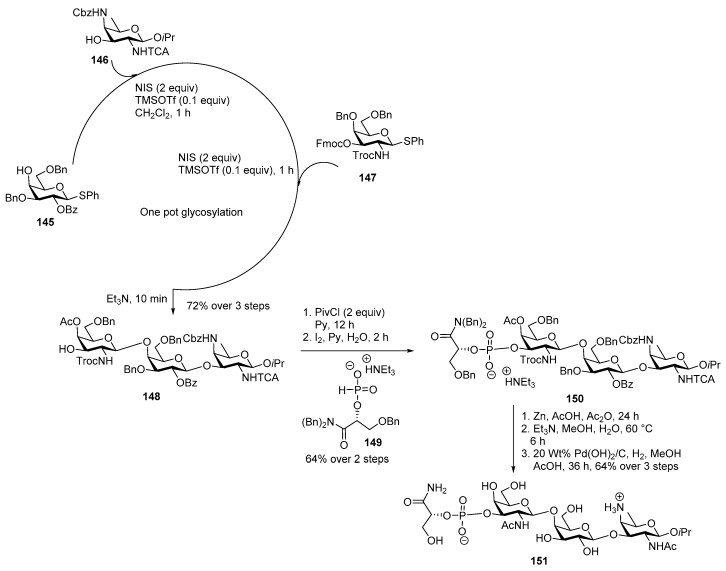
Kulkarni’s one-pot synthesis of ZPS of *P. alcalifaciens O22*.

Ovchinnikova and co-workers isolated a new phosphorylated *O*-polysaccharide from *P. alcalifaciens O22* and proposed its structure as -4)-(d-GroAN-2-P-3-)-β-d-GalNAc-(1−4)-β-d-Gal-(1−3)-β-d-Fuc-NAc4N-(1- (Scheme 16) [77].

PA O22 is a Gram negative and rod-shaped bacterium belonging to the family of Enterobacteriaceae. This pathogen is particularly known to cause diarrhea in children, travelers [78,79] and mainly involved in pericarditis [80], endocarditis [81], meningitis [82], and ocular [83] infections. These species are isolated from sputum, urine, perineum, axilla, stool, blood, and wound specimens of humans as well as from other animals and from soil and water sources [84]. 

The *O*-glycan shows a zwitterionic character because of phosphate group and free amino group of AAT residue. *P. alcalifaciens O22* polysaccharide repeating unit has a number of synthetic challenges. The main difficult tasks for the synthesis of trisaccharide are the construction of orthogonally protected rare sugar AAT building block and phosphorylation of the secondary alcohol adjacent to the amide functionality in the d-glyceramide unit. Kulkarni and co-workers reported first total synthesis of trisaccharide **151** in one-pot manner in highly regioselective fashion [85]. Coupling of 4-OH thiophenyl galactoside donor **145** and 3-OH AAT acceptor **146** using NIS, TMSOTf promoter led to the formation of 4′-OH disaccharide as a single α isomer. Addition of galactose donor **147** in the same pot in the presence of NIS, TMSOTf cleanly delivered fully protected trisaccharide. When Et_3_N was added in the same pot, Fmoc group was cleaved to afford 3-OH′′ trisaccharide **148** in 72% yield over three steps, in a one pot manner. H-phosphonate **149** was further coupled with trisaccharide **148** in the presence of pivaloyl chloride and pyridine followed by oxidation with I_2_ to furnish the phosphorylated trisaccharide **150** in 64% yield over two steps. In global deprotection first, NHTCA and NHTroc were reduced to NHAc by using Zn, AcOH, and Ac_2_O, followed by hydrolysis of esters using Et_3_N, MeOH, H_2_O at 60 °C and concomitant hydrogenolysis using H_2_, Pd(OH)_2_/C, and a drop of AcOH in MeOH to obtain target molecule **151** in 64% yield over three steps.

## *Staphylococcus aureus* Type 5 Capsular Polysaccharide

Serotype 5 and 8 capsular polysaccharides predominate among 12 known serotypes of the *S. aureus* capsular polysaccharides [86]. *Staphylococcus aureus* type 5 causes skin and soft tissue infections (SSTIs) which can lead to invasive disease with bacteremia, sepsis of endocarditis [87]. The capsular polysaccharide of *S. aureus* type 5 is a causative agent of infections in newborns, surgical patients, and immunocompromised individuals [88]. The chemical composition of both serotypes CP5 and CP8 are →4)-β-d-Man*p*NAcA-(1→4)-α-l-Fuc*p*NAc(3-OAc)-(1→3)-β-d-Fuc*p*-NAc-(1→ and →3)-β-d-Man*p*NAcA(4-OAc)-(1→3)-α-l-Fuc*p*-NAc-(1→3)-β-d-Fuc*p*NAc-(1→, respectively [89]. Both the serotypes share a common disaccharide which only differs in the acetyl ester position and an anomeric linkage. It has been reported that a bivalent vaccine of *S. aureus c*apsular polysaccharide types 5 and 8 conjugated to *Pseudomonas aeruginosa* exotoxin A (rEPA) gave 60% protection, but failed to provide long-term protection for end stage renal disease patients, who are often affected by these infections [90,91]. The major challenges in the synthesis of both trisaccharides are efficient synthesis of rare monosaccharides, such as d- and l-fucosamines, stereocontrolled installation of glycosidic linkages having a 1,2-cis-configuration including a *N*-acetyl-β-d-mannosaminuronic acid (β-d-Man*p*NAcA) and *N*-acetyl-α-l-fucosamine (α-l-Fuc*p*NAc) glycosides and having to retain acetyl esters in the target compounds.

**Scheme 17:**
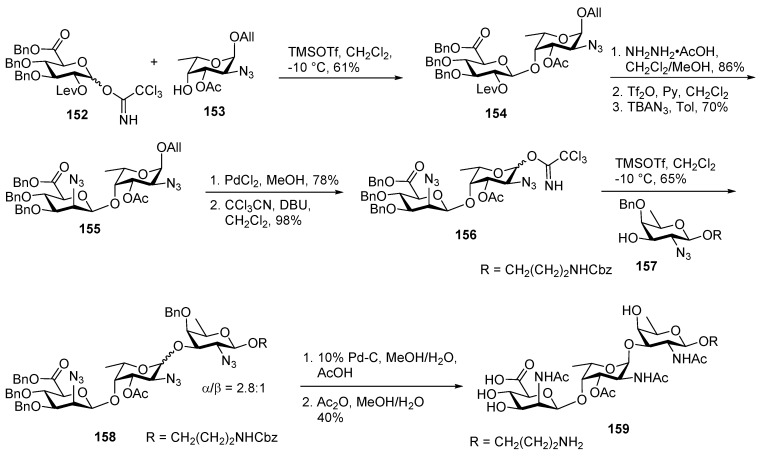
Adamo’s first total synthesis of *Staphylococcus aureus* type 5 repeating unit.

Adamo and co-workers reported the first total synthesis of *Staphylococcus aureus* type 5 in 2012 as described in Scheme 17 [92]. They employed a glucuronate donor for glycosylation and subsequent gluco to manno epimerization in the disaccharide to construct the key disaccharide which was glycosylated with the rare sugar d-fucosamine. Accordingly, disaccharide **154** was assembled via TMSOTf promoted glycosylation of glucorunic imidate donor **152** and fucosamine 4-OH acceptor **153** at −10 °C. The levulinoyl group was orthogonally deprotected using hydrazine acetic acid, followed by triflation of free 2′-OH and C2 inversion by TBAN_3_ to fashion the β-manno derivative **155** in 70% yield over 2 steps. PdCl_2_ catalyzed anomeric deallylation, followed by conversion of the corresponding hemiacetal to its imidate donor **156** and its subsequent coupling with fucosamine 3-OH acceptor **157** in the presence of TMSOTf at −10 °C afforded trisaccharide **158** as a diastereomeric mixture (α/β = 2.8:1). The α-isomer was separated from the unwanted β-isomer at this stage and was subjected to global deprotection. Azide groups were converted to acetamide and simultaneous deprotection of trisaccharide **158** was carried out by hydrogenation with 10% Pd-C, followed by treatment with acetic anhydride in MeOH to furnish target molecule **159**. 

**Scheme 18:**
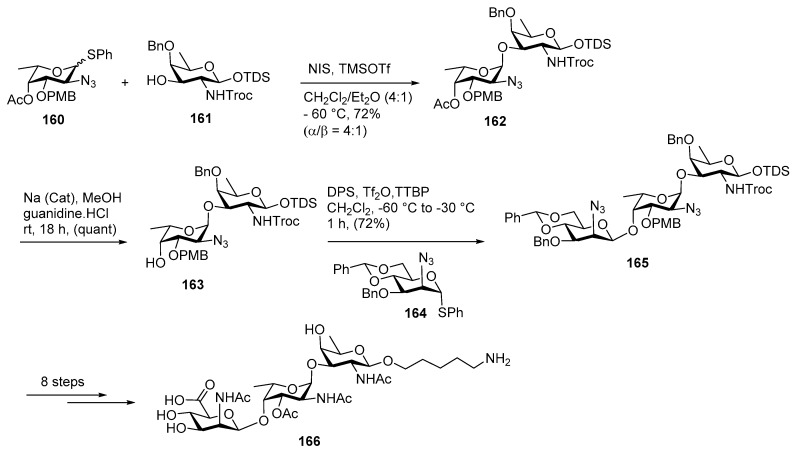
Boons’ total synthesis of *Staphylococcus aureus* type 5 repeating unit.

In 2015, Boons and co-workers reported second synthesis of *Staphylococcus aureus* type 5 polysaccharide (Scheme 18) [93]. Their strategy involves assembly of the glycan from the reducing end to the non-reducing end and a direct β-mannosylation using a mannosyl donor and subsequent late stage oxidation. The assembly of l-fucosamine donor **160** and d-fucosamine 3-OH acceptor **161** in the presence of NIS, TMSOTf as promoter and Et_2_O as participating solvent afforded α-linked glycoside **162** in 72% yield with (α/β = 4:1) ratio. The α-isomer was separated by using silica gel column chromatography at this stage. The acetyl ester was removed using NaOMe, MeOH to provide disaccharide acceptor **163** which was further glycosylated with mannosamine donor **164** using DPS/Tf_2_O promoter to deliver β-mannoside product **165** in an excellent yield of 72%. The orthogonally protected trisaccharide **165** was subsequently coupled with amino propyl linker at the late stage and further global deprotection provided target molecule **166** in good yield.

**Scheme 19:**
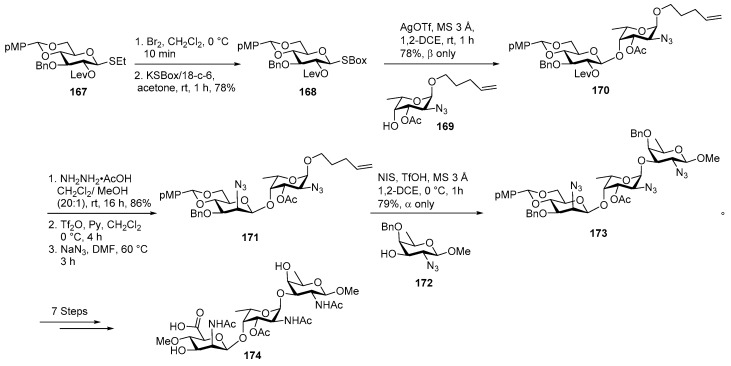
Demchenko’s total synthesis of *Staphylococcus aureus* type 5 repeating unit.

Recently, in 2015, Demchenko and co-workers reported third total synthesis of CP5 serotype polysaccharide by taking advantage of the best of both routes, as shown in Scheme 19 [94]. Notably, this route did not encounter any α*/*β mixtures in glycosylation with the rare sugar. Thioethylglycoside **167** was treated with Br_2_, CH_2_Cl_2_ at 0 °C and converted to its corresponding glycosyl bromide which upon treatment with KSBox in the presence of 18-crown-6 in acetone yielded building block **168** in 78% yield over 2 steps. AgOTf promoted orthogonal glycosylation of glucose donor **168** with l-fucosamine 4-OH acceptor **169** delivered *n*-pentenyl disaccharide **170** in 78% yield as a β isomer. In disaccharide **170**, C-2′ stereocenter was epimerized to give its corresponding manno configured derivative. For that purpose, O2-levulinoyl group was selectively cleaved using NH_2_NH_2_.AcOH, followed by triflation using Tf_2_O, pyridine and treatment with NaN_3_ in DMF at 60 °C afforded **171** in 81% yield over 2 steps.

d-fucosamine 3-OH acceptor **172** was coupled with disaccharide donor **171**, by activation of *O*-*n*-pentenyl leaving group in the presence of NIS, TfOH as a promoter in 1,2-DCE at 0 °C to obtain desired trisaccharide **173** in 79% yield as α anomer. Global deprotection and functional group modification was carried out to obtain target trisaccharide **174**. 

**Scheme 20:**
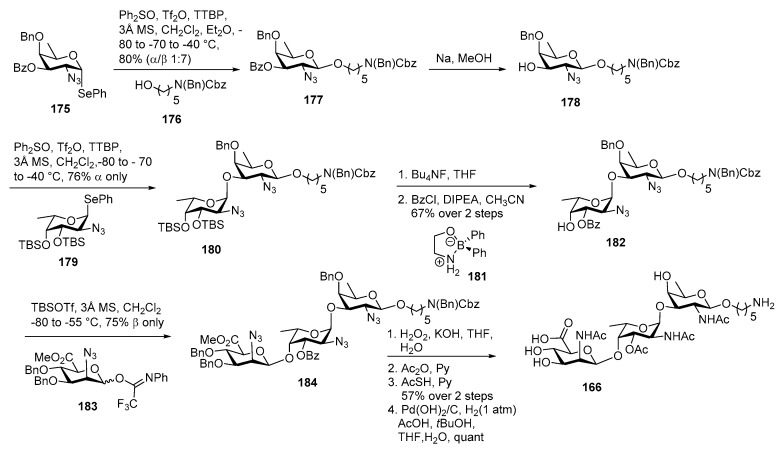
Codée’s total synthesis of *Staphylococcus aureus* type 5 repeating unit.

In 2017, Codée and co-workers reported fourth total synthesis *S. aureus* type 5 CPS repeating unit as outlined in Scheme 20 [95]. Galactosamine donor **175** was coupled with linker derivative **176** in presence of Ph_2_SO, Tf_2_O condition to afford desired product **177** in 80% yield as a diastereomeric mixture (α/β = 1:7) ratio. Benzoate group was removed using Zemplén condition to afford acceptor **178** in good yield. For the construction of α-glycosidic linkage between the l-FucN_3_ and d-FucN_3_ moieties, the more reactive 3,4-di-*O*-TBS donor **179** was glycosylated with acceptor **178** in the presence of Ph_2_SO, Tf_2_O at lower temperature to obtain disaccharide **180** as a single anomer in 76% yield. TBS ethers were removed, followed by regioselective benzoylation of the C3-O′ position, using Taylor’s diphenylborinate catalyst [96] **181** to give disaccharide acceptor **182** in 67% yield over two steps.

For the introduction of the mannosaminuronic unit, 2-azidomannuronate donor **183** was activated by TBSOTf and coupled with disaccharide acceptor **182** at lower temperature to furnish fully protected trisaccharide **184** in 75% yield as a β anomer. Functional group deprotection was carried out sequentially to deliver target molecule **166**. 

## *Staphylococcus aureus* Type 8 Capsular Polysaccharide

Demchenko and co-workers reported the first total synthesis of *Staphylococcus aureus* type 8 capsular polysaccharide in 2015 [97]. It is a Gram positive, cluster forming, bacteria and one of the highest among all bacterial pathogens [98]. It causes infection in surgical patients, trauma and burn patients, patients receiving an implant, newborns, and dialysis patients with high mortality rates frequently ensuing. 

**Scheme 21:**
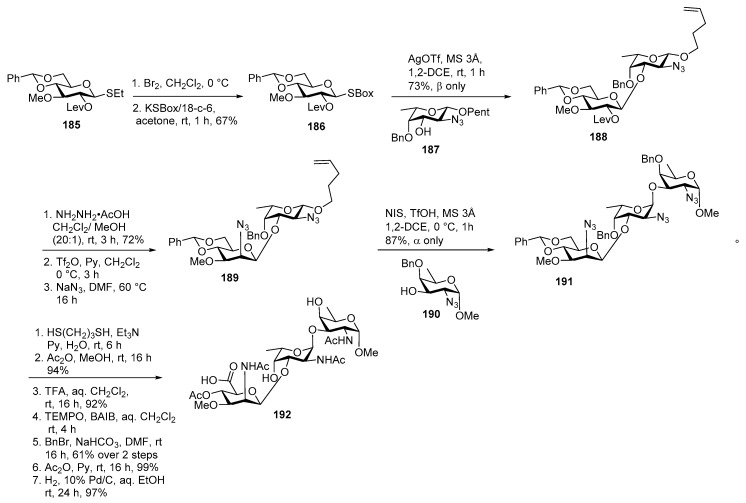
Demchenko’s total synthesis of *Staphylococcus aureus* type 8 repeating unit.

As shown in Scheme 21 ethane thiodonor **185** was converted to its glycosyl bromide upon treatment with Br_2_, CH_2_Cl_2_, followed by reaction with KSBox to convert into its glucose SBox donor **186** in 67% yield over 2 steps. SBox group was activated by AgOTf in 1,2-DCE at rt to orthogonally glycosylate donor **186** with l-fucosamine 3-OH acceptor **187** to afford *n*-pentenyl disaccharide **188** as a sole product. Then C-2′ stereocenter was inverted by selective levulinoyl group deprotection, followed by triflation of free OH and inversion with NaN_3_, DMF at 60 °C to furnish disaccharide **189** in excellent yield.

Then the corresponding disaccharide donor **189** was activated by NIS, TfOH and coupled with d-fucosamine 3-OH acceptor **190** to produce α linked trisaccharide **191** in 87% yield. The target trisaccharide **192** was accomplished by sequential deprotection of functional groups. 

## *Staphylococcus aureus* Strain M Capsular Polysaccharide

**Scheme 22:**
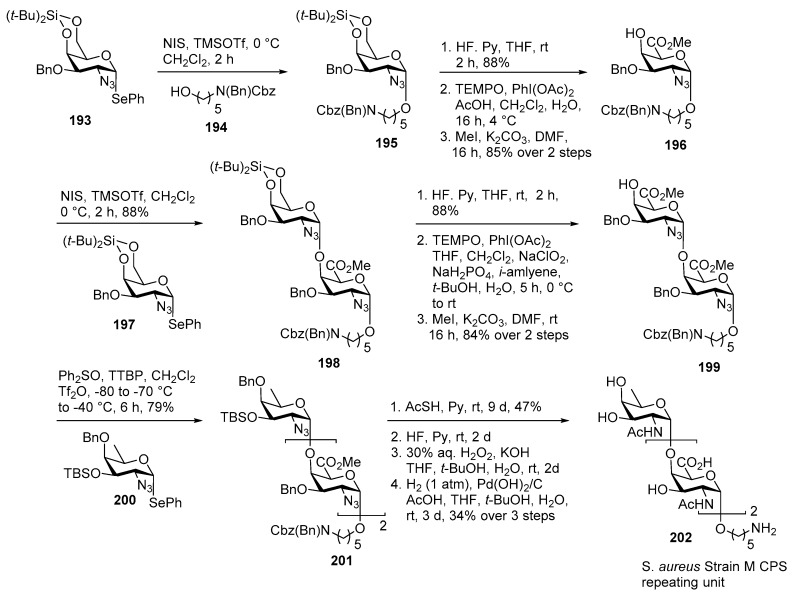
Codée’s total synthesis of *Staphylococcus aureus* Strain M repeating unit.

*Staphylococcus aureus* is a Gram-positive pathogen which is implicated in the infections of the skin, lungs, and joints and can cause life-threatening conditions such as endocarditis or toxic shock syndrome [99]. The capsular polysaccharide (CPS) consists of rare *N*-acetylgalactosaminuronic acid (GalNAcA) and *N*-acetylfucosamine (FucNAc) units [100]. The synthetic challenges are installation of cis-glycosidic linkages and synthesis of the rare monosaccharide derivatives. In 2017, Codee and co-workers reported the first total synthesis of *Staphylococcus aureus* Strain M capsular polysaccharide as delineated in Scheme 22 [99].

Glycosylation of selenoglycoside donor **193** with aminopentanol **194** proceeded smoothly, leading to the expected α-linked product **195** exclusively. Removal of the di-tertiary butyl silyl (DTBS) group, using HF in pyridine, followed by regioselective oxidation of the primary alcohol using the TEMPO/PhI(OAc)_2_ system furnished acceptor **196** in 85% over 2 steps. Coupling of DTBS protected donor **197** with acceptor **196** in the presence of NIS, TMSOTf promoter furnished disaccharide **198** in 88% yield as a sole anomer. The α-selectivity is a result of the well-known steric effect offered by DTBS group. DTBS group was removed using HF in pyridine, further primary alcohol was oxidized by using one-pot TEMPO/PhI(OAc)_2_–Pinnick oxidation protocol to give disaccharide acceptor **199** in good yield.

The final glycosylation was performed using azido-fucoside donor **200** with disaccharide acceptor **199** using Ph_2_SO/Tf_2_O as promoter at a lower temperature to give fully protected trisaccharide **201** in 79% yield with complete stereoselectivity. Deprotection of the *S. aureus* strain M repeating unit commenced with the AcSH-mediated conversion of the azides to their corresponding acetamido units, next the TBS ether was removed using HF in pyridine, and the methyl esters were saponified to give the corresponding diacid. Finally, catalytic hydrogenolysis of the benzyl carbamate and ethers provided fully deprotected target trisaccharide **202** in 34% yield over 3 steps.

## *Neisseria meningitidis* Pilin Glycans

*Neisseria meningitidis* is a Gram negative and round shaped bacterium. Meningitis is a highly contagious disease which involves inflammation of the protective membranes (meninges) of the brain and spinal cord [101]. It causes a life-threatening sepsis called meningitidis in case of children and young adults and has a high mortality rate. Thus, novel and more effective vaccines are required to control the periodic outbreak of this deadly disease. In 1995, Stimson and co-workers isolated a pilin attached trisaccharide and proposed its structure as Gal-(β1→4)-Gal(α1→3)-2,4-diacetimido-2,4,6-trideoxyhexose. Using mass spectrometry, they inferred that this trisaccharide contains a rare sugar called 2,4-diacetamido-2,4,6-trideoxyhexose (DADTH) which is α linked to l-serine but couldn’t define its stereochemistry at C-4 [102]. Kulkarni and co-workers reported the first total synthesis of both (C4 axial ‘DATDG’ and C4 equatorial ‘Bacillosamine’) the α-l-serine linked trisaccharide of *N. meningitidis* [103,104]. The major synthetic challenges for construction of this trisaccharide are synthesis of rare, deoxyamino glycans (bacillosamine and DATDG) and incorporation of two consecutive α glycosidic bonds.

**Scheme 23:**
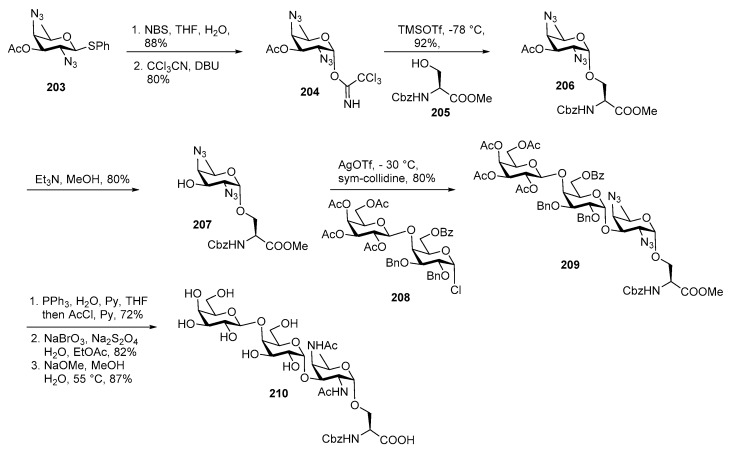
Kulkarni’s total synthesis of DATDG containing pilin glycan of *N. meningitidis.*

Total synthesis of DATDG containing trisaccharide is outlined in Scheme 23, Coupling of DATDH donor with primary alcohol of amino acid in a stereoselective fashion is a difficult task. As shown in Scheme 23, thioglycoside derivative **203** was converted to its corresponding hemiacetal upon treatment with NBS, THF, H_2_O and the so formed hemiacetal was treated with trichloroacetonitrile and DBU to afford imidate donor **204** in good yield.

Glycosylation of imidate donor **204** with l-serine derived acceptor **205** in the presence of TMSOTf promoter, and THF as a participating solvent at −78 °C cleanly afforded α linked product **206** in 92% yield. Then acetate group was selectively removed upon treatment with Et_3_N, MeOH to deliver acceptor **207** in excellent yield. Glycosyl chloride **208** was activated by AgOTf at −30 °C and coupled with acceptor **207** to furnish trisaccharide **209** in 80% with clean selectivity. Global deprotection was done in 3 steps, involving Staudinger reduction of azides, followed by *N*-acetylation, then oxidative debenzylation and de-*O*-acetylation to afford the target molecule **210** in 51% overall yield.

**Scheme 24:**
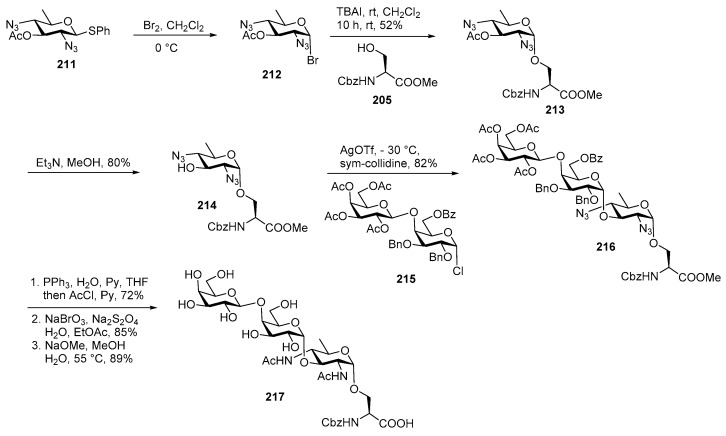
Kulkarni’s total synthesis of Bac containing pilin glycan of *N. meningitidis.*

For the synthesis of the other trisaccharide [104,105], the bacillosamine building block **211** was converted to the corresponding glycosyl bromide in situ and treated with acceptor **205** in the presence of TBAI to obtain α linked product **213** in 52% yield (Scheme 24). In this case the in-situ anomeriazation conditions offered clean α-selectivity whereas solvent participation was not very effective. The acetate group was cleaved to afford acceptor **214** which was further coupled with glycosyl chloride **215** in the presence of AgOTf at −30 °C to produce trisaccharide **216** in 82% yield.

Similar global deprotection steps carried out as previous trisaccharide furnished target trisaccharide **217** in good yield.

## *Bacillus cereus* Ch HF-PS

*Bacillus cereus* is an aerobic, Gram-positive, spore-forming bacterium [101]. Recently, Guérardel and co-workers isolated *B. cereus* ATCC 14579 and proposed its structure as →6)-Gal(α1−2)(2-R-hydroxyglutar-5-ylamido)Fuc2NAc4N(α1−6)GlcNAc(β1→, [106]. *B. cereus* can cause serious opportunistic infections such as wound infections, bacteremia, septicaemia, meningitis, pneumonia, infections of the central nervous system, endocarditis, pericarditis, respiratory infections, and peripheral infections [107,108,109,110]. It is also associated with keratitis, panopthalmitis, and other ocular ophthalmic infections which usually result in the loss of the eye [111,112,113]. *B. cereus* infections are difficult to treat, as the bacterium is resistant to several antibiotics including penicillin, ampicillin, cephalosporins, and trimethoprim [114]. The synthesis of this trisaccharide is more attractive because of its unique structure and immunogenic potential. The major synthetic challenges for constructing this trisaccharide are synthesis of the orthogonally protected rare sugar AAT building block and construction of two consecutive 1,2-cis linkages.

**Scheme 25:**
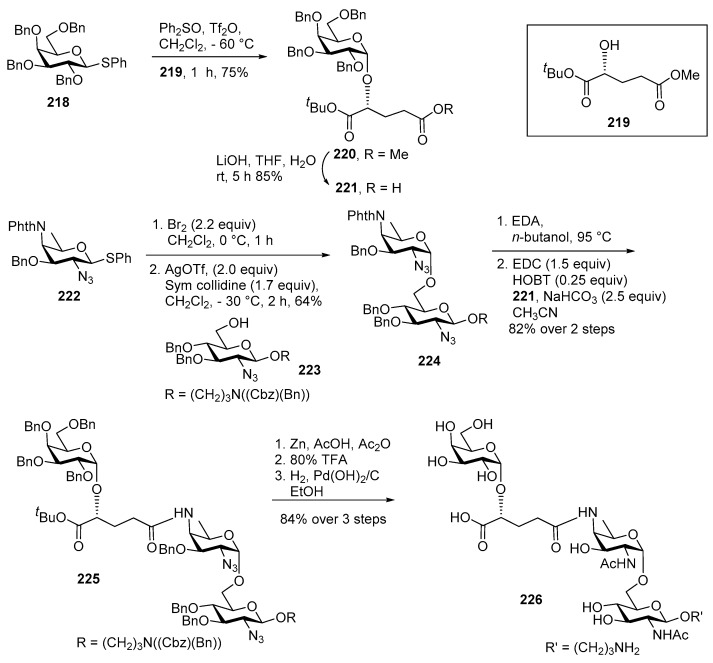
Kulkarni’s first total synthesis of *Bacillus cereus* Ch HF-PS.

Kulkarni and co-workers reported its first total synthesis in 2014 [115]. Trisaccharide **226** was assembled in [2 + 1] glycosylation manner as outlined in Scheme 25. Galactose thio donor **218** was activated by Ph_2_SO, Tf_2_O, at −60 °C and coupled with d-glutaric derivative **219** to furnish α linked product **220** in 75% yield. Then methyl ester was selectively hydrolyzed under mild basic condition to afford acid **221** in excellent yield. For the synthesis of the reducing part disaccharide, first AAT donor **222** was converted to its glycosyl bromide in situ and then treated with glucosamine 6-OH acceptor **223** in the presence of AgOTf promoter at −30 °C, to afford disaccharide **224** as a single anomer. Here the α-stereoselectivity is probably arising due to the possible formation of a more reactive β-glycosyl triflate using AgOTf and its subsequent S_N_2 type displacement by the nucleophilic 6-OH acceptor **223.** Then phthalimide group was selectively cleaved using ethylene diamine in *n*-BuOH at an elevated temperature to obtain free amine which was subsequently coupled with acid **221** using EDC.HCl, HOBT to give the fully protected trisaccharide **225** in 82% yield over 2 steps. Global deprotection via reduction of azide to NHAc, hydrolysis of *tert*-butyl esters, followed by removal of benzyl ethers using H_2_/Pd(OH)_2_ furnished the target trisaccharide **226**. 

## Glycan of *Yersinia enterocolitica*

**Scheme 26:**
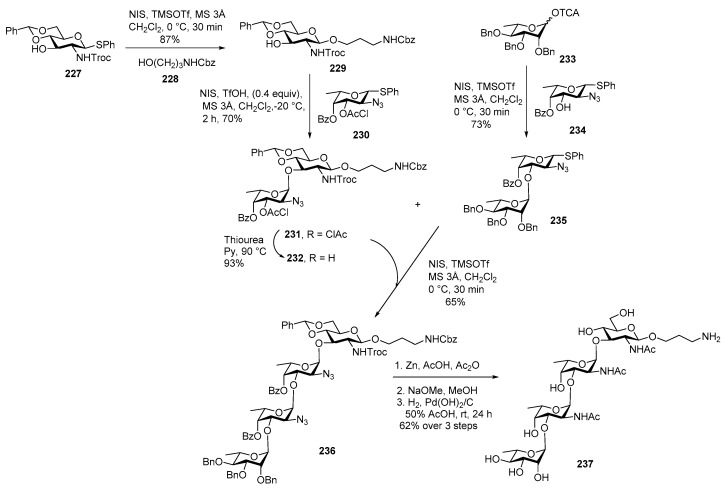
Kulkarni’s total synthesis of glycan of *Yersinia enterocolitica.*

It is a Gram negative bacteria from species of *Yersinia* genus [116]. It mainly causes enterocolitis, acute diarrhoea, mesenteric lymphadentis and pseudoappendicitis [117]. This polysaccharide was isolated in 2012 [118] and the structure of its repeating unit was elucidated as →2)-α-l-Rhap-(1→3)-α-l-FucpNAc-(1→3)-α-l-FucpNAc-(1→3)-β-d-GlcpNAc-(1 [116]. The key challenges encountered in the synthesis of this tetrasaccharide are synthesis of appropriately protected l-fucosamine building block and installation of consecutive α-linkages. The synthesis of tetrasaccharide was accomplished in [2 + 2] glycosylation route by Kulkarni and co-workers (Scheme 26) [119]. Regioselective glycosylations of glucosamine donor **227** and linker derivative acceptor **228** using NIS, TMSOTf promoter at 0 °C afforded glucosamine 3-OH acceptor **229** in 87% yield. This acceptor was further coupled with l-fucosamine donor **230** in the presence of NIS, TMSOTf promoter at −20 °C to cleanly afford α linked product **231** in 70% yield. The clean α-selectivity in this case can be attributed to the remote participation of axial C4-OBz group. The chloroacetyl group was selectively removed using thiourea in pyridine at 90 °C to afford disaccharide acceptor **232**. For the synthesis of the non-reducing end disaccharide, imidate donor **233** was coupled with l-fucosamine 3-OH acceptor **234** in the presence of TMSOTf to give **235** in 73% yield. Crucial coupling of disaccharide donor **235** and acceptor **232** in the presence of NIS, TMSOTf in CH_2_Cl_2_ delivered **236** in 65% yield. Global deprotection of tetrasaccharide **236** was achieved in 3 steps. Conversion of azide and NHTroc to NHAc using Zn, AcOH, Ac_2_O, debenzoylation using NaOMe, MeOH and debenzylation and benzylidine deprotection using H_2_/Pd(OH)_2_ smoothly delivered target tetrasaccharide **237**.

## *P. chlororaphis* Subsp. Aureofaciens Strain M71 Glycan

This compound was isolated from the root of a tomato plant by the mild acid hydrolysis of the lipopolysaccharide from *P. chlororaphis* subsp. aureofaciens strain M71 and its structure was elucidated as →2)-α-l-Rha*p*-(1→3)-α-l-Fuc*p*NAc-(1→3)-β-l-Qui*p*-NAc-(1→ [120].

**Scheme 27:**
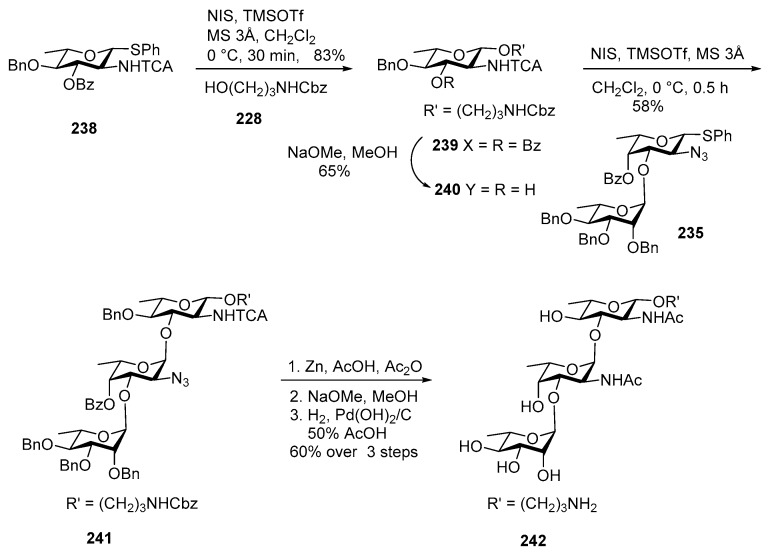
Kulkarni’s synthesis of *P. chlororaphis* subsp. aureofaciens strain M71 glycan.

Scheme 27 describes synthesis of trisaccharide **243** [119]. Quinovosamine **238** was glycosylated with amino acceptor **228** to give coupling product **239** in 83% yield. Debenzoylation of **239** using NaOMe, MeOH furnished acceptor **240** in good yield. The same non-reducing end disaccharide **235** was used for coupling with acceptor **240** by using NIS, TMSOTf promoter to afford fully protected trisaccharide **241** in 58% yield. Global deprotection accomplished in similar manner to afford trisaccharide **242**. Although compound **242** may not be useful for vaccine development the approach can be utilized for synthesizing similar l-sugar containing bacterial glycans.

## *Plesiomonas shigelloides* Serotype 51 Aminoglycoside Trisaccharide Antigen

*Plesiomonas shigelloides* is a Gram-negetive bacterium and a potential useful vaccine for travellers of subtropical and tropical regions [121]. It mainly involves in a variety of extra-intestinal infections such as sepsis, meningitis in case of children and patients with underlying diseases and causes high mortality rate [122]. This bacterium is also a common cause of severe travellers’ diarrhea [122,123]. The proposed structure of this trisaccharide was [→4)-β*-*d-Glc*p*NAc3NHbA-(1→4)-α-l-Fuc*p*Am3OAc-(1→3)-α-d-Qui*p*NAc-(1→] [123]. This oligosaccharide contains rare functional groups i.e., *N*-acetyl substituted aminodideoxyhexoses, *O*-acetyl substituted diaminodideoxyuronic acid, rare acetamidino (Am) and d-3-hydroxybutyryl (Hb) groups [123]. 

**Scheme 28:**
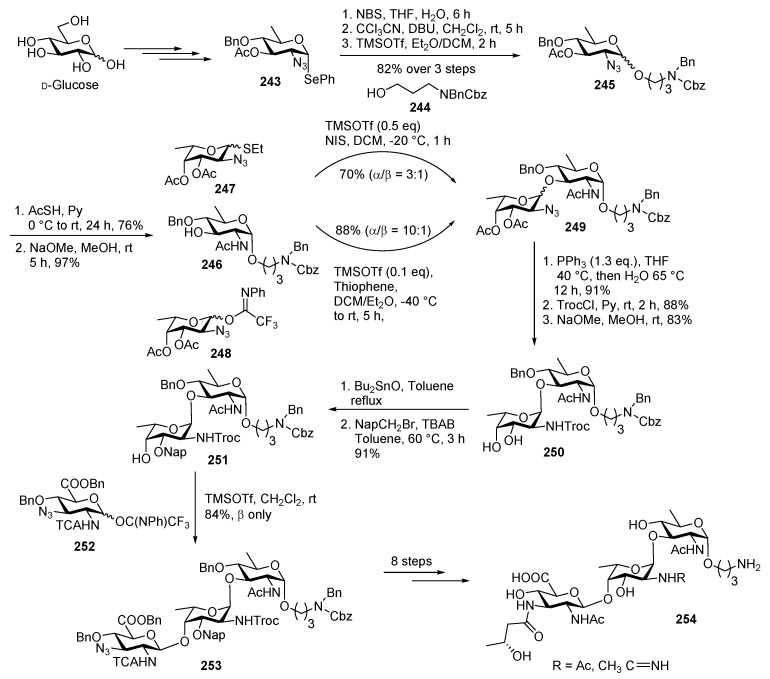
Synthesis of *P. shigelloides* serotype 51 aminoglycoside trisaccharide antigen.

Very recently, Seeberger, Yin and co-workers reported the first total synthesis of the target glycan [124]. The d-quinovosamine building block **243** was synthesized from commercially available d-glucose by a multistep route. As shown in Scheme 28, quinovosamine donor **243** was converted to its corresponding hemiacetal and treated with trichloroacetonitrile and DBU to give imidate which was subsequently coupled with linker acceptor **244** using TMSOTf promoter to afford linker derivative product **245** in 82% yield over 3 steps. Then azide was reduced to NHAc, and acetyl group was selectively removed using NaOMe, MeOH to afford quinovosamine acceptor **246** in 97% yield. Coupling of thioglycoside donor **247** using NIS, TMSOTf promoter with acceptor **246** at −20 °C led to an α/β mixture of disaccharide **249** in a ratio of 3:1. TMSOTf promoted glycosylation of imidate donor **248** in the presence of blended solvent system containing CH_2_Cl_2_, Et_2_O, and thiophene afforded disaccharide **249** with better stereoselectivity in a ratio of 10:1. Azide was reduced to amine using Staudinger method, at elevated temperature and the free amine was trocylated using TrocCl, Py. The acetate groups were removed using Zémplen deacetylation condition to give diol **250** in 83% yield. Then 3-OH was protected with napthylmethyl group using tin chemistry to deliver disaccharide acceptor **251** in 91% yield over 2 steps. Trisaccharide **253** was furnished upon glycosylation of imidate donor **252** with disaccharide acceptor **251** in 84% yield with complete β streoselectivity. Global deprotection of all functional group was carried out over several steps to accomplish target molecule **254** in good yield. 

## *Pseudomonas aeruginosa* 1244 Pilin

**Scheme 29:**
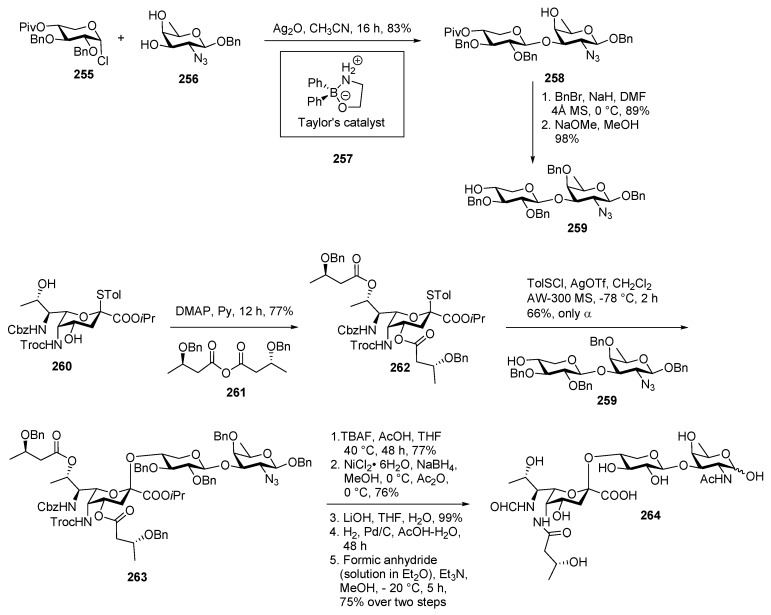
Li’s synthesis of *Pseudomonas aeruginosa* 1244 Pilin glycan.

Pseudaminic acid (Pse)-containing glycans and glycoconjugates play critical roles in bacteria [125,126]. Thus, the development of an efficient synthesis of Pse and its derivatives is of great value. *P. aeruginosa* 1244 pilin is a Gram-negative pathogen and well known for its antibiotic resistance and biofilm formation [127]. This bacteria has been shown to play an important role in immunogenicity. Cystic fibrosis type of infection in immunocompromised patients caused by *P. aeruginosa* is life threatening [128]. The structure of the pilin glycan **264**, was elucidated by Castric et al. as α-5NβOHC47NFmPse-(2→4)-β-Xyl-(1→3)-FucNAc in 2001 [129]. The pilin in *P. aeruginosa* 1244 is glycosylated with trisaccharide **264** at the C-terminal Ser148 residue through β-glycosidic linkage to the d-fucosamine [130]. In 2017, Li and co-workers reported the first total synthesis of *P. aeruginosa* 1244 pilin trisaccharide **264** through α-selective glycosylation of the Pse glycosyl donors as outlined in Scheme 29 [131]. They used *de novo* approach starting from l-allo threonine for the synthesis of pseudaminic acid and its relative functionalized derivatives. 

Disaccharide **258** was obtained by using Taylor’s glycosylations method [132]. 2-aminoethyl diphenylborinate **257** catalyzed the glycosylation of xylosyl chloride **255** and d-fucosamine acceptor **256** regioselectively and stereoselectively to give disaccharide **258** with absolute β (1→3) linkage in 83% yield. Free 4′-OH was benzylated, followed and the pivaloyl group was cleaved in the presence of NaOMe, MeOH to afford acceptor **259** in 98% yield. For the synthesis of Pse derivative **262**, the 3-benzyloxy butyrate group was installed onto both O4 and O8 of diol **260** simultaneously using the corresponding anhydride **261** in the presence of pyridine and DMAP in 77% yield. Pse glycosyl donor **262** was coupled with disaccharide acceptor **259** in the presence of TolSCl, AgOTf promoter in CH_2_Cl_2_ at −78 °C to provide the desired **263** only as axial anomer in 66% yield. Functional group deprotection was carried out in step wise manner via (1) TBAF-mediated Troc deprotection, (2) O4-to-N5 acyl transfer, (3) azide to amine reduction using a nickel boride reagent, (4) in situ acetylation by Ac_2_O, and (5) mild saponification to obtain an acid which was treated with H_2_ and Pd/C in HOAc–H_2_O to remove the Cbz group and five benzyl groups, and the desired formyl group was next installed onto the released N7 by freshly prepared formic anhydride at −20 °C to deliver final product *P. aeruginosa* 1244 pilin trisaccharide **264**.

## Summary and Outlook

Bacteria contain rare deoxy amino sugars which are absent in host cells. This difference in their glycan structures can be exploited for vaccine development. The rare sugars which are present in bacteria are however not available commercially. Tremendous progress has been achieved in recent years toward the development of novel protocols for procurement of the rare sugar building blocks and their stereoselective assembly to synthesize structurally complex bacterial glycans. These advances offer pure and structurally well-defined and linker-attached glycans which are ready for conjugation with carrier proteins. Recent advances in one-pot and automated assembly of glycans [133] would is expected to expedite procurement of bacterial glycans. The immunological studies carried out with these glycoconjugates are expected to lead to identification of new epitopes for vaccine development.
